# Anaphylactic reaction to the first dose of subcutaneous methotrexate in JIA: a case report and literature review

**DOI:** 10.1186/1546-0096-12-S1-P200

**Published:** 2014-09-17

**Authors:** Leonardo Campos, Renata Sobral, Vivian Almeida, Rozana Almeida, Marise Lessa, Christianne Diniz, Marta Rodrigues, Sheila Oliveira, Flavio Sztajnbok

**Affiliations:** 1 Pediatric Rheumatology, Instituto de Pediatria e Puericultura Martagao Gesteira (IPPMG), Federal University of Rio de Janeiro, Brazil; 2 Pediatric Rheumatology, Adolescent Health Care Center, State University of Rio de Janeiro (NESA/UERJ), Rio de Janeiro, Brazil

## Introduction

Methotrexate is the most used DMARD drug in the treatment of juvenile idiopathic arthritis and its efficacy has been demonstrated for more than 20 years. The usual dose is 10 to 15mg/m^2^/week. It is a safe and well-tolerated drug by children and the most common side effects are gastrointestinal, hematologic and liver toxicity.

## Objectives

To report a case of a 13-years old adolescent girl diagnosed with polyarticular JIA that had an anaphylactic reaction to the first dose of subcutaneous methotrexate.

## Methods

Case report and literature review.

## Results

A 13-years old adolescent girl diagnosed with polyarticular JIA (RF +/ANA-/HLA-B27-) received the first dose of subcutaneous methotrexate in our Day Care centre due to refractoriness to NSAIDs. While she was leaving the building after 10 minutes of medication she developed urticaria, angioedema, shortness of breath and hypoxia (O_2_ saturation of 85%). After administration of 0.5ml of 1:1000 adrenaline, 500ml of saline, 2.5mg of dexchlorpheniramine and 125mg of methylprednisolone she recovered and was discharged home after a short period of stay. The methotrexate she used was also for intrathecal administration and had only distilled water as suspension.

## Conclusion

This is the first case report of anaphylaxis to methotrexate in a patient with JIA without previous exposure. Considering it is a very rare and fatal reaction, we suggest that the first subcutaneous dose should be administrated in a health center and the patient should wait 30 minutes before leave.

## Disclosure of interest

None declared.

